# Pseudo-wound infection after a caesarean section: Case report of unrecognized Pyoderma Gangrenosum

**DOI:** 10.1016/j.ijscr.2020.03.041

**Published:** 2020-04-13

**Authors:** Carlina E. van Donkelaar, Johanna M.H. de Haan, Johan F.M. Lange, Marjolijn de Vries, Barbara Horváth

**Affiliations:** a Department of Obstetrics and Gynecology, Ziekenhuisgroep Twente, Almelo, the Netherlands; b Department of Surgery, University Medical Center Groningen, Groningen, the Netherlands; c Department of Dermatology, University Medical Center Groningen, Groningen, the Netherlands

**Keywords:** C-section, caesarean section, NPWT, negative pressure wound therapy, PG, Pyoderma Gangrenosum, Pyoderma gangrenosum, Abdominal necrosis, Caesarean section

## Abstract

**Background:**

Pyoderma Gangrenosum (PG) is a rare auto-inflammatory disease, characterized by painful ulcerative skin-lesions often developing at sites of injury or surgery because of the typical pathergy phenomena. We describe an unusual case of PG after a caesarean section with excessive extra-cutaneous manifestation within internal organs.

**Presentation of case:**

A 21-year-old Dutch primigravida developed signs of sepsis after a caesarean section. Despite antibiotic treatment, fast clinical deterioration occurred. Exploration of the wound showed necrosis of the uterus and surrounding tissues. Due to the progression of necrosis, consecutive debridement procedures were executed resulting in a substantial abdominal wall defect. The progressive clinical course of the necrosis combined with absence of positive wound cultures and histology of prominent interstitial neutrophilic infiltration, led to the diagnosis ‘Pyoderma Gangrenosum’. Treatment with high dose corticosteroids led to rapid regression of the disease. After several weeks, the abdominal wall defect was surgically corrected under systemic corticosteroid therapy.

**Discussion:**

This case of PG is unique due to the excessive extra-cutaneous presentation, which contributed to delayed diagnosis. Several surgical interventions in the active stage of disease resulted in expansion of PG and substantial morbidity for the patient.

**Conclusion:**

Post-operative PG can mimic infectious diseases, but treatment is substantially different. This case of extensive PG highlights the importance of timely recognition and treatment of the disease to reduce iatrogenic morbidity.

## Highlights

• Pyoderma Gangrenosum (PG) is a rare auto-inflammatory disease.
• It can develop after surgery due to the pathergy phenomena.
• Consider PG in the differential diagnosis of a suspected surgical wound infection.
• Avoid surgical treatment as long as the disease is active to reduce morbidity.

## 1 Introduction

Pyoderma Gangrenosum (PG) is a rare neutrophilic dermatosis, closely related to auto-inflammatory diseases. The incidence rate is approximately 3–10 patients per million per year, with a peak incidence between 20 and 50 years and women more commonly affected [1,2].

Typically, PG lesions develop at sites of injury caused by trauma or surgery, also known as the pathergy phenomena. Due to the high inflammatory load, PG is often mistaken for severe bacterial infection, such as necrotizing fasciitis. Limited knowledge of PG amongst physicians, other than dermatologists, contributes to delayed diagnosis and treatment.

We describe an atypical case of PG after a caesarean section (C-section) with extra-cutaneous involvement affecting the internal organs, in which delayed diagnosis attributed to iatrogenic damage and a high morbidity of the disease. This case-report has been reported in line with the SCARE criteria [3].

## 2 Case

**Fig. 1:**
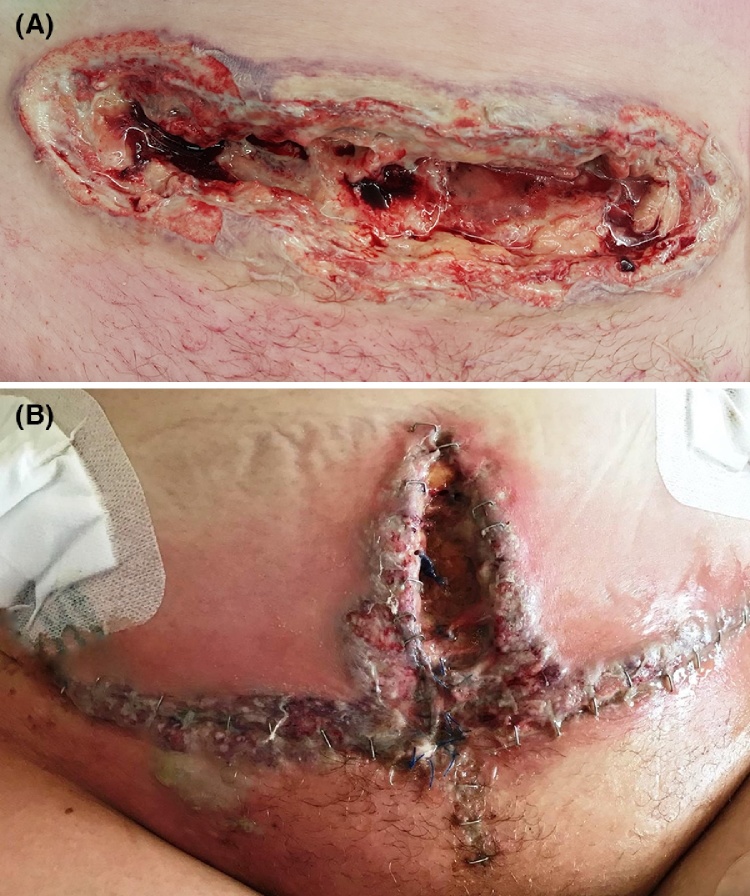
A: The C-section wound with pustular edges and violaceous borders (7th day postpartum). B: The abdominal wound dehiscence after multiple debridement’s (19th day postpartum). Wound edges are violaceous with a pustular bulla on the lower right abdomen, surrounded by diffuse erythema.

The 21-year old patient underwent an uncomplicated emergency C-section at 32 + 1 weeks due to preterm labor and breech position. The pregnancy was uncomplicated so far, and her medical history reported no relevant information. Two days postpartum, she developed fever (39.5 degrees Celsius) in combination with increased CRP of 236 (normal value <10) and leukocytosis (35.9 10^9^/L, normal value 4–10 × 10^9^/L). Physical examination revealed pain in the lower abdominal region with an enlarged uterus. Ultrasound excluded retained placental fragment. Antibiotic treatment was started under the diagnosis of endometritis. Multiple cultures (blood, wound and vaginal) remained negative. On 5th day postpartum, the C-section wound showed purulent discharge and wound dehiscence. Partial opening of the wound confirmed an intact abdominal fascia (Fig. 1a). Wound therapy was started and antibiotic treatment continued.

Lack of clinical improvement necessitated a CT-scan which revealed discoloration of the anterior uterus wall, suspect for necrosis. Soon afterwards, the patient developed systemic inflammatory response syndrome with acute kidney failure. Treatment was initiated according to the sepsis protocol and she was admitted to the intensive care unit (ICU). Exploratory laparotomy showed purulent necrotic tissue on the lower uterine segment of the uterus (between the uterus and bladder), along the abdominal muscles, and the retroperitoneal tissue surrounding the ureters. Extensive debridement was performed. Due to necrosis progression two additional laparotomies with debridement were performed. Still, all blood and wound cultures remained free of meaningful pathogens. Additional serology blood tests ruled out human immunodeficiency virus, syphilis and systemic lupus erythematosus. On 19th day post-partum, the abdominal wound edges once again showed dehiscence with signs of necrosis (Fig. 1b). After the third debridement, a negative pressure wound therapy (NPWT) system was applied to cover the abdominal wall defect. Additionally, two paracolic drains were placed intra-abdominally.

**Fig. 2:**
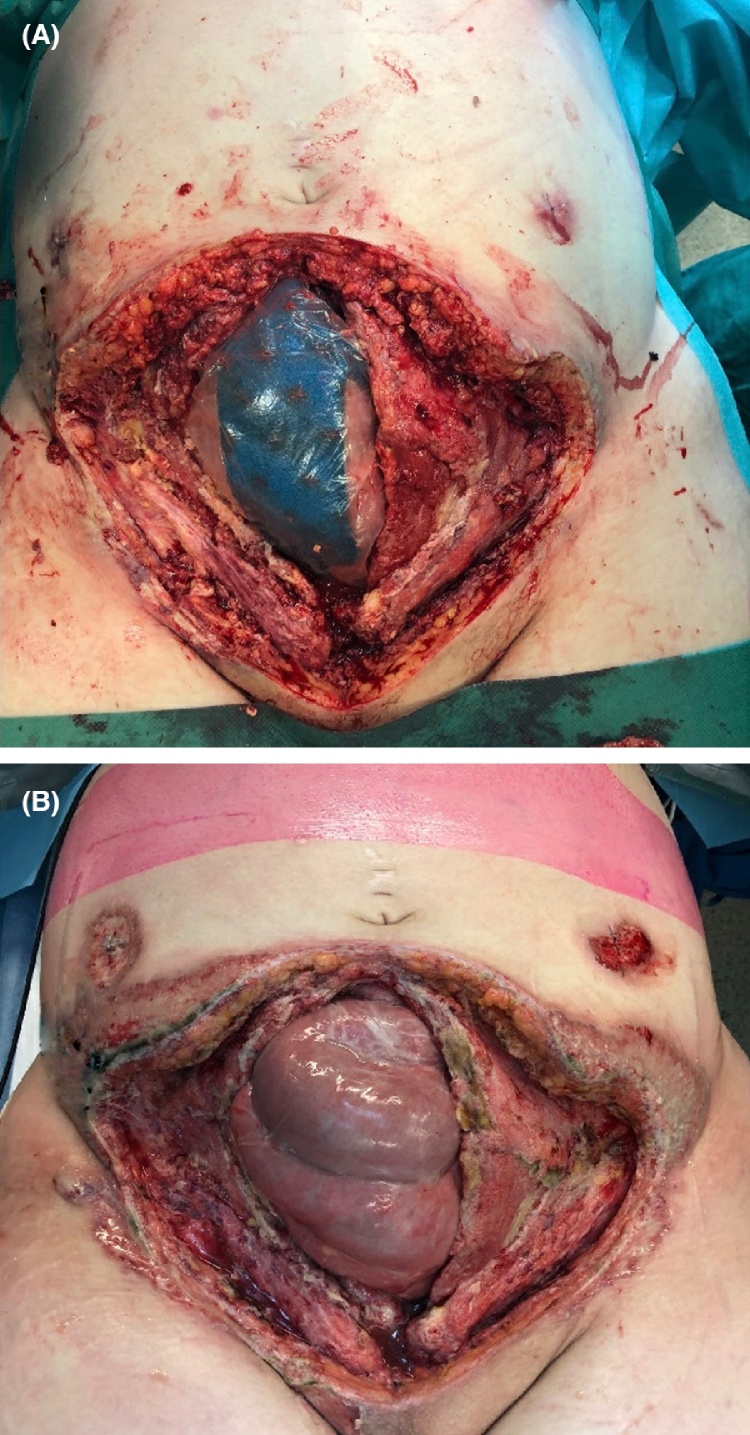
A: The abdominal wound with NPWT-system in situ (20th day postpartum). B: New ulceration around the left and right paracolic drains is visible (22nd day postpartum).

The complexity of the case and progression of illness required transfer to an academic hospital (20 days postpartum). Explorative laparotomy showed a substantial abdominal wall defect with necrotic wound edges which were debrided. A new NPWT-system was placed on the wound. The drain entry wounds showed no signs of inflammation (Fig. 2a). However, two days later, the drain entry wounds showed newly formed purulent ulcers (Fig. 2b), which were not related to the necrotic tissue around the abdominal wound. A dermatologist was consulted and biopsies were taken, showing prominent interstitial neutrophilic infiltration with a differential diagnosis of infection, Sweet’s syndrome and PG. The combination of the clinical course, progression under surgical and antibiotic treatment and absence of meaningful pathogens in the cultures, led to the diagnosis of PG.

**Fig. 3:**
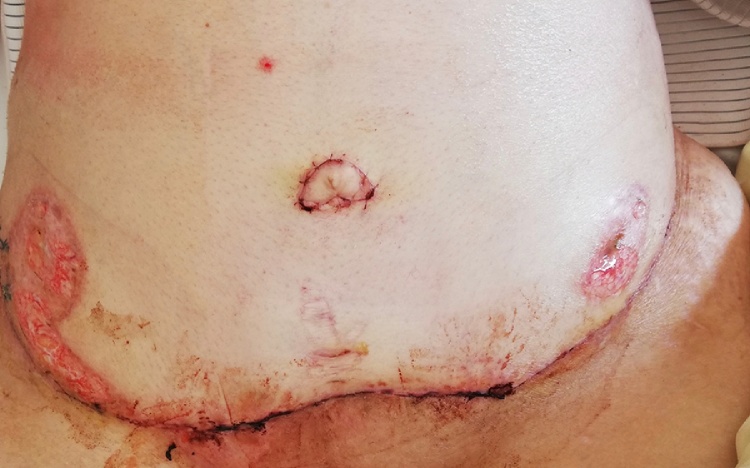
The abdomen after abdominoplasty, 3 months postpartum.

Immediate treatment with high-dose systemic corticosteroids (Prednisolone 1 mg/kg) led to rapid clinical improvement. Treatment was later expanded with a T-cell inhibitor (Cyclosporine-A 5 mg/kg), and the corticosteroid dose reduced. Twelve weeks’ post-partum, the patient underwent abdominoplasty (Fig. 3) under immunosuppressive treatment. Immunosuppressive treatment was tapered off over several months and has since been stopped. Several follow-ups have showed no signs of re-activation of PG.

## 3 Discussion

The exact underlying pathophysiology of PG is unknown, but involves dysregulation of the immune response. Patients have significant overexpression of cytokines and chemokines, and there is evidence of gene mutations in several auto-inflammatory genes [1]. More than 50% of the patients have an associated systemic disease, most commonly inflammatory bowel disease (Crohn’s Disease and Colitis Ulcerosa). Other related diseases are arthritis and specific hematological malignancies [1,2]. In this patient, a thorough work-up for associated conditions remained negative.

Although diagnostic criteria have been proposed by Maverakis et al. [2], PG remains difficult to diagnose. The typical skin lesion of this subtype is a painful ulcerating wound with a violaceous, elevated/undermined border, often progressively spreading peripherally. The lesions are accompanied by spiking fever and general malaise and laboratory findings include high CRP and leukocytosis [1,4].

PG develops in 25–50% of the cases after surgery or trauma to the skin due to neutrophil activation, the so-called pathergy phenomen [3,4]. PG is often described after bowel surgery (most commonly located parastomal) and breast surgery [1,5], but rarely after C-sections. Interestingly, many of the cases described in literature, report a premature C-section due to preterm labor or suspected fetal distress [[6], [7], [8], [9], [10]]. An association with PG and premature delivery has not yet been established.

PG after surgery is often mistaken for a bacterial wound infection with subsequent delay in appropriate treatment. In a case-series of 36 PG patients, 29 patients were misdiagnosed as wound infection and 13 patients received debridement of the lesions [11]. Remarkable in this case, is that the PG appeared largely intra-abdominal instead of intra-cutaneous, which increased the suspicion of an infectious disease such as necrotizing fasciitis, and further delayed diagnosis. Extra-cutaneous manifestation of PG is rare, a recent review reported 96 cases described between 1973 and 2018 [12]. Of these cases, pulmonary manifestation was the most common, but genital and cervical manifestations of PG have also been reported. There have been no previous reports suggesting uterine manifestation of PG, so this case may be the first, although it remains difficult to determine if the PG was actually initiated intra-uterine, or whether it expanded from the skin into the internal organs.

Treatment of PG is based on local or systemic immune suppression, depending on the clinical course [1]. For systemic treatment, high-dose prednisone is the preferred choice, with rapid effect. Surgery with active PG must be avoided as aggravates the surrounding tissue and leads to PG progression [1]. In this case, the extensive abdominal debridement’s during the active phase of the disease might contributed to the destructive course.

After the diagnosis, the NPWT-system was successfully used to bridge the period of active PG and after the PG was stabilized, the abdomen could be closed. NPWT under immune suppression is no routine treatment of PG wounds, but a few described cases present successful use in treatment of deep tissue wounds related to PG [13]. NPWT is known for increasing tissue perfusion, enhancing cellular proliferation, and reducing bacterial load [14]. Furthermore, in case of PG it seems to result in significant pain reduction [13].

Long-term outcome of patients with PG remains unpredictable, even after effective treatment. Recurrence rates up to 70% are described, but are based on small numbers of patients [4]. In patients with a history of PG, prophylactic administration of perioperative immunosuppressive therapy is recommended in case of future surgical procedures.

Despite advances in diagnostics and treatment, PG is still associated with high morbidity and potential mortality. In this case, the delayed diagnosis certainly contributed to the high morbidity. This patient underwent many laparotomies at young age, with large consequences: the abdominal muscles were largely affected and her uterus was extirpated. Furthermore, because of the long stay on the ICU and surgical ward, she was not able to take care of her newborn and adequately bond. Luckily, she recovered relatively well and upon today no recurrence of the disease occurred.

## 4 Conclusion

Pyoderma gangrenosum is a rare neutrophilic dermatosis that can occur after surgery because of the pathergy phenomena. The clinical symptoms can mimic a secondary bacterial wound infection, consequently, the disease is often not recognized and mistreated. Timely recognition and adequate treatment of the disease is of utmost importance to avoid iatrogenic morbidity.

## Declaration of Competing Interest

No conflict of interest is to declare.

## Sources of funding

Funding from the University of Groningen, the Netherlands, was received for publication of this article.

## Ethical approval

Our local institutional board decided that no ethical approval is necessary for this case-report.

## Consent

Written informed consent was obtained from the patient for publication of this case report and accompanying images.

## Author contribution

CED and JMHH drafted the manuscript.

JFM, MV and BH were involved in treatment of the patient and reviewed the manuscript.

All authors read and approved the final manuscript.

## Registration of research studies

Name of the registry: Not applicable.

Unique identifying number or registration ID: NA.

Hyperlink to your specific registration (must be publicly accessible and will be checked): NA.

## Guarantor

CE van Donkelaar is the guarantor.

## Provenance and peer review

Not commissioned, externally peer-reviewed.
